# Multi-lineage emergence of USA300 MRSA in in Chile and a potential role for environmental copper resistance

**DOI:** 10.64898/2026.10.22.761065

**Published:** 2026-10-23

**Authors:** Amy Elizabeth Campbell, José Rodrigo W. Martínez, Lorena Díaz, Colleen Bianco, Erin Theiller, Swetha Rajagopal, Alexander Arvanitis, Alejandra Londoño Jiminez, Ahmed M. Moustafa, Lina Rivas, Patricia García, Cesar A. Arias, José M. Munita, Paul J. Planet, the MICROB-R Consortium

## Abstract

Methicillin-resistant *S. aureus* (MRSA) is prevalent worldwide, but individual strains of MRSA tend to spread and expand in relatively confined geographic areas. The USA300 lineage of MRSA is most well-known for its rapid epidemic spread in North America during the first decade of the 2000’s leading to an overall increase in serious skin infections and the emergence of methicillin resistance in the majority of community acquired *S. aureus* infections. A distinct but closely related South American epidemic clade of MRSA USA300 (USA300-SAE), first reported in 2006, proliferated rapidly in Northern South America but was rarely found in other Latin American countries. In a new survey of 274 invasive MRSA isolates from 2022 collected in Chile, we saw a marked increase in USA300-SAE in Southern South America. We found that Chilean USA300-SAE is phylogenetically diverse and was likely introduced multiple times from multiple different South American countries over the course of more than a decade, but only recently became a highly prevalent clone. Using novel molecular diagnostic methods for isolates collected in 2023, we estimate that USA300-SAE made up more than 22% of invasive MRSA strains in Chile in that year and is likely still increasing. We show that copper-associated genes in the signature copper and mercury resistance mobile element (COMER) of USA300-SAE are strongly conserved, and that isogenic mutants of the copper resistance genes in COMER are highly susceptible to copper killing at concentrations seen in tap water. These findings suggest that environmental exposure to copper may provide a fitness advantage that enhances spread of this lineage.

